# Engineered Bacteria of MG1363-pMG36e-GLP-1 Attenuated Obesity-Induced by High Fat Diet in Mice

**DOI:** 10.3389/fcimb.2021.595575

**Published:** 2021-02-25

**Authors:** Lingfang Wang, Tingtao Chen, Huan Wang, Xiaoli Wu, Qing Cao, Ke Wen, Ke-Yu Deng, Hongbo Xin

**Affiliations:** ^1^ National Engineering Research Center for Bioengineering Drugs and the Technologies, Institute of Translational Medicine, Nanchang University, Nanchang, China; ^2^ College of Basic Medicine, JiangXi University of Traditional Chinese Medicine, Nanchang, China

**Keywords:** glucagon-like peptide 1 (GLP-1), genetically engineered bacteria, fatty acid oxidation, peroxisome proliferator-activated receptors (PPARα), MG1363-pMG36e-GLP-1

## Abstract

**Background:**

Although gut hormone glucagon-like peptide 1 (GLP-1) has been widely used for treating diabetes, the extremely short half-life greatly limits its application. The purpose of this study is to explore the effects of an engineered bacteria with expression of GLP-1 on obese mice induced by high fat diet (HFD).

**Methods:**

The engineered strain of MG1363-pMG36e-GLP-1 (M-GLP-1) was constructed and its anti-obesity effects were evaluated *in vivo*. The bodyweight, the morphology of adipose and liver tissue, and liver function were examined. Quantitative RT-PCR and Western blot were used to measure the expressions of the genes involved in fatty acid oxidation synthesis. The intestinal microbial diversity was detected with high-throughput sequencing analysis.

**Results:**

The engineered bacteria could produce GLP-1. It also significantly decreased the bodyweight and improved the glucose intolerance in the obese mice induced by HFD. Moreover, the strain also reduced the triglyceride (TG) in serum, protected liver, as well as decreased the intracellular TG in liver tissues of the obese mice. Furthermore, our results showed that the expressions of the genes including peroxisome proliferator-activated receptors α (PPARα) and its target genes were enhanced in liver tissues when mice treated with M-GLP-1. Finally, we found that the engineered strain markedly increased intestinal microbial diversity.

**Conclusion:**

Our results suggested the genetically engineered bacteria that constitutively secreted GLP-1 could improve obesity and the mechanism may be related to promoting fatty acid oxidation and increasing intestinal microbial diversity of the obese mice.

## Background

Obesity is a metabolic syndrome resulting from multiple factors, such as diet and environment. The morbidity of obesity is dramatically increasing since 1980 and is the main cause resulting in other metabolic diseases, such as type 2 diabetes, cardiovascular disease and non-alcoholic fatty liver disease (NAFLD). It has been reported that people with high BMI (>25 kg/m^2^) was associated with the high cardiovascular diseases (Collaborators et al., 2017). Obesity is prone to insulin resistance and is characterized by an ectopic fat accumulation. Excessive triglyceride (TG) accumulation in adipose tissue is one of the main causes of obesity. The content of TG is regulated by synthesis and degradation. Besides that, triglyceride is formed by long-chain fatty acids and glycerol, and the fatty acids are mainly synthesized in liver and their degradation are mainly caused by fatty acids β oxidation. During obesity, it is often found that the expressions of genes related to fatty acid oxidation are significantly decreased.

PPARs and SREBPs are the most important transcription factors in glucose and lipid metabolism whose abnormality are more easily to lead to obesity and other metabolic diseases (Pawlak et al., 2015; Wang et al., 2017; Wang et al., 2018). Among these transcription factors, PPARα plays the key role in fatty acid metabolism (Michalik et al., 2006). Studies indicated that the activation of PPARα up-regulated the expressions of genes involving in various aspects of fatty acid metabolism including peroxisomal and mitochondrial fatty acid β-oxidation, which eventually promoted the fatty acids uptake, utilization, and catabolism (Kersten, 2014). PPARα is involved in biological functions *via* affecting the expressions of many target genes. And previous studies showed that many target genes were regulated by PPARα, including classical target genes of Pyruvate dehydrogenase lipoamide kinase isozyme 4 (PDK4), Acyl-Co A Oxidase 1 (ACOX1), and carnitine palmitoyl transferase 1α (CPT1α) (Kersten, 2014; Pawlak et al., 2015). Moreover, although the host genetics, metabolism, lifestyle, and diet can influence the development of obesity, the concrete mechanisms that lead to the disease are not to be elucidated clearly. With the development of bioinformatics, it has been reported that the intestinal microbiota had profound roles in the onset and development of obesity *via* affecting the host’s nutrients acquisition and energy homeostasis that ultimately influence the storage of lipids in fat cells (Rosenbaum et al., 2015; Dahiya et al., 2017). Many studies have demonstrated that obesity has a strong connection with the intestinal microbial diversity and composition (Torres-Fuentes et al., 2017). It has been recently reported that obesity was associated with a reduced ratio of *Bacteroidetes* to *Firmicutes* at phylum level and a reduction of *Akkermansia* (Pedersen et al., 2016; Houghton et al., 2018). In addition, recent literatures showed that the increase of *Prevotella copri* and *Bacteroides vulgatus* aggravated insulin-resistance in patients with obesity. It was reported the reduced abundance of *Proteobacteria* and *Escherichia coli*, together with the increase of *Firmicutes* were associated with NAFLD (Liu R et al., 2017; Loomba et al., 2017).

Glucagon-like peptide-1 (GLP-1) is an extensively studied anti-hyperglycemic hormone that has roles in glucose-dependent stimulation of insulin secretion. Emerging evidences indicated that GLP-1 had efficiently treated type 2 diabetes due to its action for lowering blood glucose (Drucker, 2018). In addition to its role in diabetes, GLP-1 also plays protective roles in cardiovascular diseases through its direct and indirect actions such as reducing inflammation and increasing endothelial function (Drucker, 2016). It has been reported that GLP-1 analogue could prevent non-alcoholic steatohepatitis in non-obese mice (Yamamoto et al., 2016). The roles of GLP-1 in obesity has also been reported, and it could regulate adipogenesis in 3T3-L1 preadipocytes (Yang et al., 2013). Furthermore, literature showed that GLP-1 had been approved by FDA to treat obesity, served as an anti-obesity agent mainly through reducing appetite or enhancing satiety in central nervous system (Srivastava and Apovian, 2018). Although GLP-1 shows therapeutic effects in metabolic diseases, the half-life of GLP-1 is very short and it is quickly cleaved into the inactive truncated form by dipeptidyl peptidase-4 (DPP-4) within a few minutes (Deacon et al., 1995; Marathe et al., 2013). Therefore, it is very important to extend the action time of GLP-1 through different methods.

The short half-life of GLP-1 limits its application in metabolic diseases, such as diabetes and NAFLD. In addition, the roles of GLP-1 in obesity have been not clarified clearly. In the present study, in order to determine the roles of GLP-1 in HFD-induced obesity and overcome its limitations, we constructed an engineered bacteria of MG1363-pMG36e-GLP-1 (M-GLP-1) that can constitutively secrete GLP-1 (1–37) through oral administration, and we evaluated its efficiency of alleviating obesity and potential mechanisms on obese mice induced by high fat diet (HFD) *in vivo*. Our study showed that the engineered bacteria could produce GLP-1 and significantly reduced blood glucose in type I diabetes mouse model and bodyweight of obese mice induced by HFD. The mechanism may be associated with increased fatty acid oxidation and enhanced the intestinal microbial diversity. These results suggested that the engineered bacteria of M-GLP-1 may be a potential reagent for obesity treatment.

## Methods

### Construction of the Engineered Strain

The GLP-1 sequence (1-37, XM_023217335.1) was synthesized by Kingsy Biotechnology Co. (Nanjing, China). The fragment was inserted into pMG36e, a prokaryotic expression vector (Chen et al., 2018; Fang et al., 2019) to get the plasmid of pMG36e-GLP-1. Then the plasmid was transformed into *Lactococcus lactis* MG1363 using electroporation to construct the MG1363-pMG36e-GLP-1 strain (M-GLP-1).

### Production and Detection of GLP-1

M-GLP-1 was incubated anaerobically in de Man Rogosa Sharpe (MRS) medium (Oxoid, Basingstoke, UK) at 37°C overnight and was sub-cultured three times. Then, the bacteria were cultured in MRS medium for another 48 h, and the medium was then centrifuged at 8,000 g for 10 min to obtain the supernatant. The production of GLP-1 in fermentation supernatant was measured using GLP-1 enzyme-linked immunosorbent assay (ELISA) kit (Millipore, Billerica, MA, USA).

### High Fat Diet (HFD)-Induced Obesity Mouse Model

Eight-week-old C57BL/6 male mice were purchased from Hunan Si Lake King of Experimental Animal Co., Ltd. (Changsha, Hunan, China). The mice had free access to water and were fed with either standard chow (ND; N = 5) or HFD (60% HFD, D12492; Research Diets Inc. New Brunswick, NJ, USA) *ad libitum* for 15 weeks. Then, the obesity mice were fed with PBS (HFD+PBS; N = 5), MRS medium containing 10^9^ M-GLP-1 (HFD+GLP1 group; N = 5) every 2 days for another 30 days. The study was approved by the Ethical Committee of the Second Affiliated Hospital of Nanchang University, and all methods were conducted in accordance with the approved guidelines.

### Glucose and Insulin Tolerance Test

Glucose concentrations were determined using a glucometer (OneTouch Ultra; LifeScan, Inc.) in blood collected from the tail vein at the indicated time points. For the GTT, mice were fasted for 16 h and then injected i.p. with D-glucose (1.5 g/kg). For the insulin tolerance test (ITT), 6-h-fasted mice were injected i.p. with 0.75 U/kg insulin and tail vein blood glucose was then measured at the indicated times.

### Serum Biochemistry and Intracellular Triglyceride Measurements

The triglyceride (TG), aspartate aminotransferase (AST), aspartate aminotransferase (ALT) in mice serum were tested in the Clinical Laboratory of the Second Affiliated Hospital of Nanchang University. The contents of intracellular triglyceride in the liver tissue were measured using triglyceride assay kit (PPLYGEN, Beijing, China) according to the manufacturer’s instruction, and normalized to total protein concentrations.

### Histopathological Examination

Epididymis adipose and liver tissues were isolated and fixed with 4% (v/v) paraformaldehyde and embedded in paraffin. The 5 μm sections were prepared and then rehydrated by xylene and declining grades of ethanol for 5–6 min, and then washed three times using PBS for another 5 min, and the slides were used for hematoxylin–eosin (HE) staining (Liu Q. et al., 2017).

### Total RNA Extraction and Real-Time PCR (q-PCR)

Total RNA from liver tissue was isolated using the Trizol method (Thermo Fisher) followed by DNase treatment. The RNA was reversely transcribed using the Takara high capacity cDNA synthesis kit. PCR primers were designed using Primer 5.0 software (Primer-E Ltd., Plymouth, UK) and quantitative PCR amplification was performed using an ABI-ViiA7 PCR machine (Applied Biosystems, USA). The amplification was programed to start at 95°C for 10 min, followed by 40 cycles of degeneration at 95°C for 30 s, annealing at 60°C for 34 s, and extension at 60°C for 1 min. Relative mRNA expression levels (fold change) of the target genes were analyzed by the 2^−△△Ct^ method based on q-PCR data of comparative critical threshold (Ct). The primers used in this study were listed below: PPARα, 5-GGGTACCACTACGGAGTTCACG-3 (sense) and 5- CAGACAGGCACTTGTGAAAACG-3 (anti-sense); CPT1α, 5- ACATCCCTAAGCAGTGCCAGTT-3 (sense) and 5- TCGTCCGGCACTTCTTGATC-3 (anti-sense); PGC1α, 5-CCCTGCCATTGTTAAGACC-3 (sense) and 5-TGCTGCTGTTCCTGTTTTC-3 (anti-sense); ACOX1, 5-GCCTGAGCTTCATGCCCTCA-3 (sense) and 5-ACCAGAGTTGGCCAGACTGC-3 (anti-sense); ACSL, 5-CTGGTTGCTGCCTGAGCTTG-3 (sense) and 5-TTGCCCCTTTCACACACACC-3 (anti-sense); GLUT4, 5-GGCTTTGTGGCCTTCTTTGA-3 (sense) and 5-CTGAAGAGCTCTGCCACAATGA-3 (anti-sense).

### Western Blotting

For Western blot analysis, liver tissues were lysed in RIPA buffer (0.5% NP-40, 0.1% sodium deoxycholate, 150 mM NaCl, 50 mM Tris-Cl, pH 7.5). Lysates were centrifuged at 10,000 g for 10 min at 4°C and protein concentration was determined using the Bradford reagent (Bio-Rad) with bovine serum albumin (BSA) as standard. Lysates were resolved by sodium dodecyl sulfate-polyacrylamide gel electrophoresis (SDS-PAGE), transferred to polyvinylidene difluoride (PVDF) membrane (Millipore), and probed with PPARα (mouse monoclonal antibody, Santa Cruz, Cat: sc-398394) and CPT1α (mouse monoclonal antibody, Abcam, Cat: ab128568) antibodies.

### DNA Extraction

The fresh fecal samples of mice were collected, diluted five-fold with ddH_2_O, and homogenized according to a bead-beating method. Briefly, samples were suspended in 1 ml lysis buffer containing 0.3 g sterile zirconium beads in the screw-capped tube. The tubes were bead-beaten at 8,000 g for 3 min in a mini-bead beater, following the DNA extraction using the TIANamp Genomic DNA Kit (Tiangen Biotech Co., Ltd., Beijing, China) and was stored at −20°C (Meng et al., 2017a; Meng et al., 2017b).

### High-Throughput Sequencing Analyses

The concentration and quality of extracted genomic DNA were tested prior to sequencing using the Nano-drop spectrophotometer (NanoDrop Technologies, Wilmington, USA). The extracted genomic DNA was used as the template to amplify the V3-V4 region of 16S rRNA genes using primers of 338F/806R (GenBank accession number SRP137627). PCR reactions, pyrosequencing of the PCR amplicons, and quality control of raw data were performed as described previously (Fang et al., 2016). Paired-end reads from the original DNA fragments were merged using FLASH when at least some of the reads overlap the read generated from the opposite end of the same DNA fragment, and paired-end reads was assigned to each sample according to the unique barcodes. Then, sequence analysis was performed by UPARSE software package (Uparse v7.0.100, http://drive5.com/uparse) using the UPARSE-OTU and UPARSE-OTUref algorithms. In-house perl scripts were used to analyze alpha (within samples) and beta (among samples) diversity. Sequences with ≥97% similarity were assigned to the same operational taxonomic units (OTUs). Sequence was picked as a representative for each OTU, and the ribosomal database project (RDP) classifier was used to annotate taxonomic information for each representative sequence. Cluster analysis was preceded by weighted UniFrac distance using quantitative insights into microbial ecology (QIIME) software package, and the species found to be differentially abundant were characterized for their metabolic capacity by PICRUSt, Version 1.0.0 (Javurek et al., 2017).

### Data Analysis

Statistical analysis was performed with Prism software version 6.02 (Graph Pad Software, San Diego, CA, USA). Data are presented as means ± SD. Statistical significance was evaluated by one-way or two-way analysis of variance (ANOVA) followed by Tukey’s multiple comparison tests. Statistical significance was set at *p < 0.05, **p < 0.01, ***p < 0.001.

## Results

### M-GLP-1 Strain Significantly Reduced Body Weight in Obese Mice Induced by High Fat Diet

**Figure 1:**
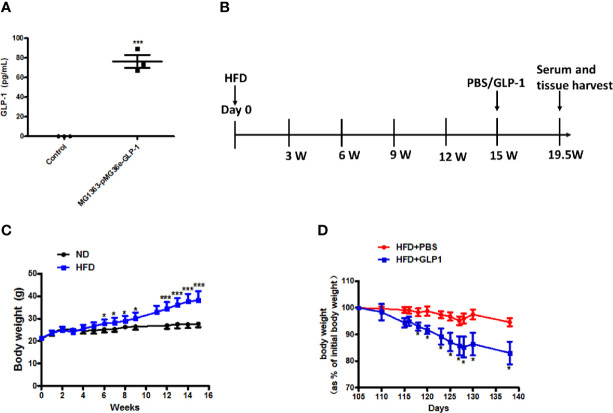
Effects of the engineered strain of MG1363*-*pMG36e-GLP-1 (M-GLP-1) on body weight in high fat diet mice. **(A)** Expressions of GLP-1 in bacteria. The *Lactococcus lactis* MG1363 were transformed with prokaryotic expression vector pMG36e-GLP-1 or pMG36e (control) and the fermentation supernatant were collected for detection of the GLP-1 levels using an Elisa Kit after the bacteria were cultured overnight. **(B)** Experimental schematic in mice. The hyperglycemia/obesity mouse model was induced by high fat diet (HFD) for evaluating the therapeutic effects of the engineered strain of M-GLP-1. The mice were given PBS or M-GLP-1 after 15 weeks of HFD feeding, respectively. **(C)** The curves of bodyweights in the mice. The HFD mice were fed with HFD for 15 weeks and then divided into two groups at 15^th^ week for treated with PBS or GLP-1 for 1 month, respectively. **(D)** Percentage of bodyweight gain in mice. The bodyweights of the PBS and GLP-1 treated mice which were fed with HFD were measured every other day, and the percentage of the bodyweight gain was represented with the initial bodyweight of the mice at 15^th^ week HFD fed as a control. The values represent the means ± SD, *p < 0.05, and ***p < 0.001, n = 5 per group.

To verify the GLP-1 production capability of M-GLP-1 strain, the GLP-1 concentration was determined using ELISA kit *in vitro*. As shown in **Figure 1A**, the concentration of GLP-1 was about 76 pg/ml in M-GLP-1 group, and no GLP-1 was detected from the control vector group. Moreover, to evaluate the therapeutic potential of engineered bacteria in obese mice induced by high fat diet (HFD, for 15 weeks), the engineered bacteria and PBS were administered every 2 days for another 4.5 weeks. The experimental scheme is illustrated in **Figure 1B**. The results showed that HFD successfully induced obese mice (**Figure 1C**) and the percentage of the bodyweight gain of mice was markedly reduced by M-GLP-1 strain compared with control group (**Figure 1D**). Moreover, we have evaluated the effects of engineered bacteria but lacking GLP-1 on HFD-fed mice, while the engineered bacteria lacking GLP-1 failed to reduce the body weight compared with the PBS group (**Figure S1A**). Taken together, these results provided evidence that engineered bacteria could reduce body-weight in mice induced by HFD.

### M-GLP-1 Strain Remarkably Reduced Adipose Tissue Weight and Hepatic Lipid Accumulation in HFD Fed Mice

**Figure 2:**
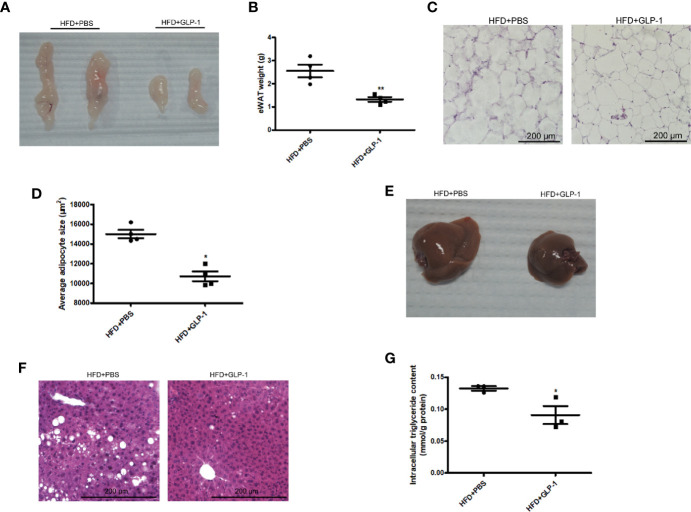
Effects of M-GLP-1 on adipose tissue weight and hepatic lipid accumulation in mice fed with HFD. **(A)** The images of epididymal adipose tissue from PBS and GLP-1 treated mice. **(B)** The weights of epididymal adipose tissue from the two groups of mice. **(C)** The morphological analysis of Hematoxylin and eosin staining in epididymal adipose tissue from the mice. **(D)** The measurement of adipocyte cross-sectional surface area from the two group mice under HFD. **(E)** The image of liver tissue from the two groups of mice. **(F)** The morphological analysis of Hematoxylin and eosin staining in liver tissue from the mice. **(G)** The intracellular TG levels in two groups of mice under HFD. The values represent the means ± SD, *p < 0.05 and **p < 0.01, n = 5 per group.

To further examine the effects of M-GLP-1 strain on obesity, the epididymal adipose tissue and liver tissue were collected from the mice fed with HFD. As shown in **Figures 2A, B**, the weight of epididymal adipose tissue from mice treated with M-GLP-1 strain was lower than control group. Moreover, the H&E staining showed that the size of adipocyte in GLP-1 group was slightly smaller than control (**Figure 2C**). In addition, the quantitative measurement of adipocyte cross-sectional surface area was consistent with the HE staining results (**Figure 2D**). We also observed that M-GLP-1 strain-treated mice displayed less hepatocyte steatosis when compared with the PBS-treated mice (**Figure 2E**). In addition, there was a significant decrease of hepatic lipid deposition in the liver from mice treated with M-GLP-1 strain compared with control mice through H&E staining (**Figure 2F**). Furthermore, the intracellular TG level was decreased in liver tissue from M-GLP-1 group (**Figure 2G**). These results demonstrated that the engineered strain reduced the weight of adipose tissue and hepatic lipid accumulation in HFD fed mice.

### M-GLP-1 Strain Improved Glucose Intolerance and Liver Functions in Mice Fed With HFD

**Figure 3:**
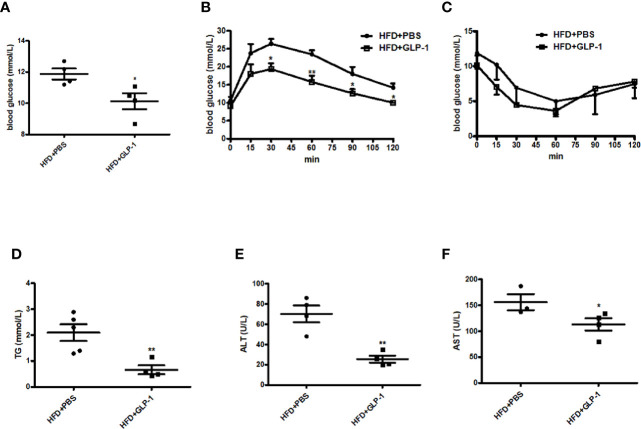
Effects of M-GLP-1 on insulin resistance and liver functions in HFD mice. **(A)** Blood glucose concentrations were measured in 16 h fasted HFD-fed mice treated with PBS or M-GLP-1 at 18^th^ week. **(B)** Glucose tolerance test was performed in mice fed with HFD at 18^th^ week. **(C)** Insulin tolerance test was performed in mice fed with HFD at 19^th^ week. Plasma triglyceride **(D)**, ALT **(E),** and AST **(F)** were measured in mice fed with HFD at 19.5^th^ week. The values represent the means ± SD, *p < 0.05 and **p < 0.01, n = 5 per group.

Obesity is often associated with insulin resistance. In this study, we also evaluated the effects of M-GLP-1 on insulin resistance and glucose intolerance in the mice fed with HFD. We found that the concentration of the fasting blood glucose was decreased in M-GLP-1 group compared with control at the 18^th^ week (**Figure 3A**). Moreover, the glucose tolerance test (GTT) showed that the glucose intolerance was improved in M-GLP-1 group (**Figure 3B**), suggesting that M-GLP-1 strain might be able to increase the glucose uptake. However, the insulin tolerance test showed no significant difference between these two groups, indicating M-GLP-1 strain may have no effect on insulin sensitivity (**Figure 3C**). Besides that, we also examined the serum lipid concentration and liver function in the two group mice and found that serum TG level was significantly decreased in GLP-1 group (**Figure 3D**). Moreover, the biomarkers of liver function including ALT and AST were reduced in M-GLP-1 strain group (**Figures 3E, F**). Taken together, all these results indicated that the engineered strain of M-GLP-1 significantly improved glucose intolerance and liver functions in mice fed with HFD.

### M-GLP-1 Strain Increased the Expressions of Genes Involving in Fatty Acid Oxidation in Obese Mice Induced by HFD

**Figure 4:**
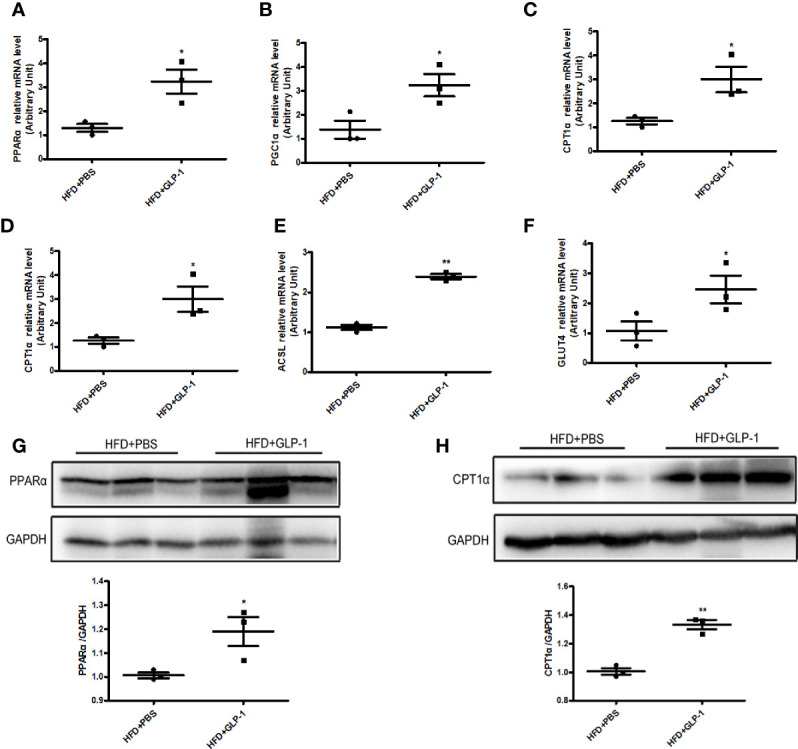
Effects of M-GLP-1 on expressions of genes involving in fatty acid oxidation in mice fed with HFD. Quantitative analysis of the mRNA expressions of PPARα **(A)**, PGC1α **(B)**, CPT1α **(C)**, ACOX1 **(D)**, ACSL **(E)**, and GLUT4 **(F)** in liver tissue from the mice treated with or without M-GLP-1. The western blot images and quantitative analysis of the protein levels of PPARα **(G)** and CPT1α **(H)** were determined in liver tissue from the mice treated with or without M-GLP-1. The values represent the means ± SD, *p < 0.05 and **p < 0.01, n = 3–5 per group.

To further explore the mechanisms of M-GLP-1 strain against obesity and reducing hepatic lipid accumulation, we first detected the expressions of genes involved in fatty acid synthesis. We found there was no significant difference in the expressions of the genes such as SREBP1, DGAT2, and p-ACC in liver tissue between M-GLP-1 group and control group (**Figure S1B**). Then we examined the expressions of genes involved in fatty acid oxidation in liver tissue. Real-time PCR analysis indicated that the expressions of PPARα, a key transcript factor that regulated fatty acid oxidation, and PGC1α which played an important role in mitochondrial biogenesis, were increased in liver tissue from M-GLP-1 strain group compared with control (**Figures 4A, B**). Moreover, we observed that the expressions of the target genes of PPARα including CPT1α, ACOX1, and ACSL were also up-regulated in M-GLP-1 strain group (**Figures 4C–E**). In addition, the expressions of glucose transporter 4 (GLUT4) were increased in M-GLP-1 group (**Figure 4F**). Consistent with the changes of mRNA, the protein levels of PPARα and its target gene CPT1α were also increased in the liver of M-GLP-1 treated mice compared with control (**Figures 4G, H**). All these results indicated that the engineered strain of M-GLP-1 attenuated obesity mainly through increasing the expressions of genes involving in fatty acid oxidation.

### M-GLP-1 Strain Increased Intestinal Microbial Diversity in Obese Mice Induced by HFD

**Figure 5:**
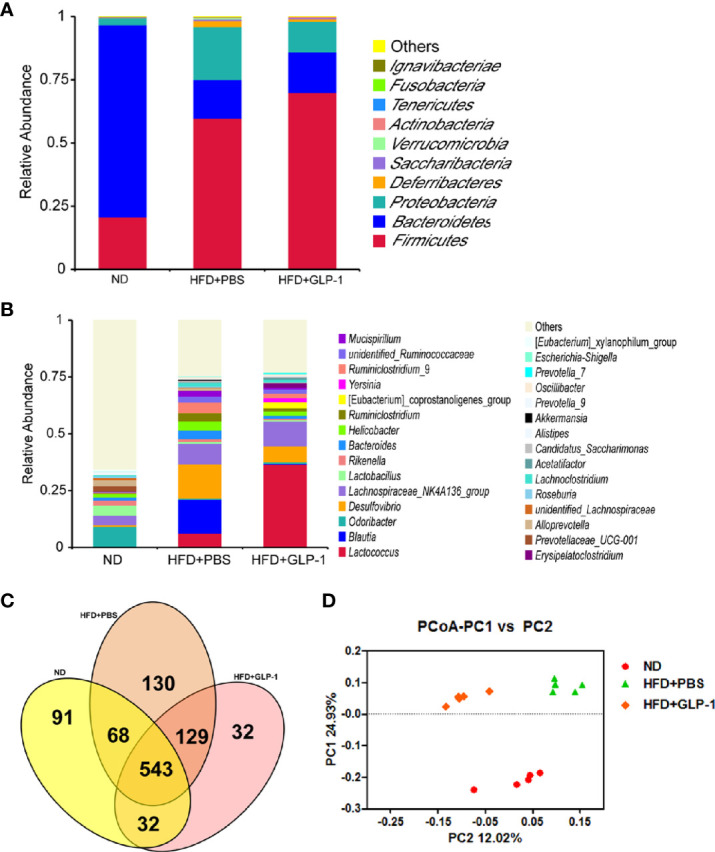
Effects of M-GLP-1 on intestinal microbial diversity in mice fed with HFD. **(A)** The relative abundance of the bacteria among the two groups at phylum level. **(B)** The relative abundance of the bacteria among the two groups at genus level. **(C)** Scalar-Venn representation of the vaginal microbiota among the two groups. **(D)** The principal co-ordinates analysis (PCoA) among the two groups. The values represent the means ± SD, n = 5 per group.

It has been reported that the alterations of intestinal micro-organisms play key roles in the obesity development (Dahiya et al., 2017), and the abundance of bacteria is beneficial to the improvement of obesity. In our study, we detected the intestinal microbes in the two groups with high-throughput sequencing methods. The valid data were filtered and those sequences with over 97% similarity were clustered as one OUT, and 959,181 filtered clean tags (63,945.40 tags/sample) and 8,665 OTUs were got from all the samples with an average of 577.67 OTUs per group. The results showed that the richness of *Firmicutes* and *Proteobacteria* was increased while the richness of *Bacteroidetes* was decreased in HFD+PBS group compared with normal diet (ND) group at phylum level (**Figure 5A**). Compared with PBS group, the richness of *Proteobacteria* was significantly reduced in M-GLP-1 group, while the richness of *Firmicutes* was further increased. In addition, there was a slight increase in the richness of *Bacteroidetes* in M-GLP-1 group compared with PBS group (**Figure 5A**). Furthermore, we found the richness of *Lactococcus* in M-GLP-1 group (36.51%) was obviously higher than that in PBS group (6.25%) at genus level (**Figure 5B**). Besides that, the Venn results indicated that the percent of common OTUs (543) was 73.98% (543/734, ND), 62.41% (543/870, HFD+PBS), 73.78% (543/736, HFD+GLP-1) (**Figure 5C**). The results from the principal co-ordinates analysis showed that ND and HFD group had a significant difference, and the samples in M-GLP-1 group were separated from the PBS group (**Figure 5D**). All these results indicated that the engineered strain of M-GLP-1 increased intestinal microbial diversity of mice and restored the disturbed microbial composition to normal level in the obese mice induced by HFD.

## Discussions

Obesity and diabetes are chronic inflammation-mediated diseases caused by environmental exposures, e.g. diet, lifestyle, poor sleep, endocrine disorder, and intestinal flora status (Swinburn et al., 2011; Javurek et al., 2017). The main cause of obesity is that the energy intake exceeds the energy expenditure and thus disrupts the body’s energy balance. Studies have shown that microbiota imbalance had a strong link with obesity (Virtue et al., 2019). It has been reported that imbalance of intestinal flora resulting in energy homeostasis imbalance ultimately resulted in obesity. It has been observed that probiotics (e.g. *Lactobacillus* spp. and *Bifidobacterium* spp.) possess anti-obesity effects *via* altering gut microbial community and production of bioactive compounds (Pedersen et al., 2016; Li et al., 2017). Moreover, the probiotics also influenced lipid metabolism and improved inflammation through reducing fat storage, altering serum lipid profiles, increasing expressions of fatty acid oxidation genes, and decreasing expressions of pro-inflammatory cytokines (Dahiya et al., 2017). In this study, we constructed the engineered strain of MG1363-pMG36e-GLP-1 (M-GLP-1) which could produce the GLP-1 (the effective glucose-lowering hormone). Our results showed that the engineered strain significantly reduced the bodyweights of the obese mice induced by HFD.

GLP-1 is the most extensively studied gut hormone for curing diabetes and its mimetic drugs were also developed to treat type 2 diabetes mellitus (T2DM). Besides that, researchers found that GLP-1 had other biological functions including the protection in nervous system and immune system, which could be helpful in improving obesity (Holscher, 2014). Recently, researchers constructed an engineered commensal bacteria which could express full-length form of GLP-1 (1-37), and they found the strain could be taken orally to improve diabetes because the intestinal cells were reprogrammed as glucose-reactive insulin-secreting cells (Duan et al., 2015). With the development of gene engineering technology, it is possible to endow some new functions to bacteria. Dinesh K *et al.* modified *Escherichia coli* Nissle to make it express genes encoding Phe-metabolizing enzymes, and it could reduce blood phenylalanine (Phe) concentration by 38% compared with the control (Isabella et al., 2018). Caroline B *et al.* engineered the oral probiotics *E. coli* Nissle 1917 to convert NH3 to l-arginine (l-arg), and their phase 1 clinical results indicated that this strain could improve the hyperammonemia disorders including urea cycle disorders and hepatic encephalopathy (Kurtz et al., 2019). In our study, the bacteria *Lactococcus lactis* MG1363 we used here was probiotics which it had advantages in terms of safety. The engineered strain markedly decreased the hepatic lipid accumulation, protected liver function, and improved glucose intolerance in the obese mice induced by HFD. Therefore, the engineered strain not only efficiently treats the obesity and the symptoms associated with obesity, but also it has no obvious side effects.

To further explore the possible mechanisms of anti-obesity of M-GLP-1 strain, we firstly examined the changes in signaling pathway that was related to triglyceride degradation. We observed that the expressions of genes which had important roles in fatty acid oxidation, such as PPARα and its target genes were up-regulated in liver tissue in GLP-1 group. It has been reported that GLP-1 and its analogue could prevent non-alcoholic steatohepatitis (Yamamoto et al., 2016). As GLP-1 has a very short half-life, the GLP-1 receptor agonists (GLP1RAs) which had longer half-lives have been developed for treating T2DM and other metabolic diseases including obesity and liver diseases (Andersen et al., 2018). It is possible for M-GLP-1 strain to provide a long-term effect for anti-obesity due to producing GLP-1 continuously. In addition, the increased expression of PGC1α which participated in mitochondrial biogenesis suggested that the energy expenditure might be increased in mice treated with M-GLP-1.

Increasing evidence indicated the obesity had a strong connection with host intestinal microbiome. It has been reported that remodeling in gut microbiota played important roles in the metabolic improvements associated with caloric restriction, including browning of fat and maintaining good glycemic control (Fabbiano et al., 2018). Microbial diversity is important to a healthy gut microbiome. The literature indicated that gut microbiota accelerated gastrointestinal motility while suppressing the expression of GLP-1 receptor in myenteric neural cells throughout the gastrointestinal tract (Yang et al., 2017). It has been reported that the microbiome influenced host metabolism, and that changes in intestinal microbial population contributed to insulin resistance and obesity. The mechanisms mainly may be involved in the following aspects including inflammation, intestinal permeability and short-chain fatty acids, and so on (Singer-Englar et al., 2019). In addition, it was reported that GLP-1 or dual GLP-1/GLP-2 receptor agonist made metabolic and gut microbiome changes. The article also pointed out the microbiome alterations may have connection with the converging biological actions of GLP-1 and GLP-2 receptor signaling on caloric intake, glucose metabolism, and lipid handling (Madsen et al., 2019). In our research, we also evaluated the roles of engineered bacteria in intestinal microbial composition using high-throughput sequencing methods. Our results suggested that HFD profoundly influenced the microbial composition both at phylum level and at gene level, and the PCoA results indicated that the microbial diversity in HFD mice was obvious different from ND and M-GLP-1 group. In addition, the richness of *Lactococcus* in M-GLP-1 group was as high as 36.51%, indicating that the *Lactococcus* could exist in mice intestine (for the taking of 10^9^ M-GLP-1 every other day) and the strain may play its beneficial roles in defending obesity.

## Conclusion

Our results showed that genetically engineered bacteria of M-GLP-1 significantly reduced blood glucose and bodyweights of the obese mice induced by HFD, demonstrating the mechanism of anti-obesity of M-GLP-1 strain may be related to promoting fatty acid oxidation and increasing intestinal microbial diversity of the obese mice. These findings suggest that genetically engineered bacteria expressed GLP-1 may have therapeutic potential for the treatment of obesity. However, the miss of vector-carrying strain and the limited number of HFD mice used in the present study hinder us to make statistically sound conclusions.

## Data Availability Statement

The extracted genomic DNA was used as the template to amplify the V3-V4 region of 16S rRNA genes using primers of 338F/806R (GenBank accession number PRJNA448831).

## Ethics Statement

The animal study was reviewed and approved by Nanchang University Institutional Animal Research Committee.

## Author Contributions

TC, HX, and LW participated in the study design. WH, XW, QC, KW, and K-YD analyzed and interpreted the data. TC, HX, and LW wrote the manuscript. All authors contributed to the article and approved the submitted version.

## Funding

This work was supported by grants from the National Natural Science Foundation of China (Nos. 31560264, 82000354, 91639106, 81873659, and 81760140), Excellent Youth Foundation of JiangXi Scientific Committee (No. 20171BCB23028), Science and Technology Plan of Jianxi Health Planning Committee (No. 20175526), and Jiangxi Provincial Department of Science and Technology (20192BAB215005, 20181BAB205009).

## Conflict of Interest

The authors declare that the research was conducted in the absence of any commercial or financial relationships that could be construed as a potential conflict of interest.

## Supplementary Material

Supplementary Figure 1 **(A)** The curves of bodyweights in the mice treated with PBS or engineered bacteria but lacking GLP-1 on HFD-fed mice in pre-experiment. **(B)** The western blot images of the protein levels of SREBP1, DGAT2, and p-ACC were determined in liver tissue from the mice treated with or without M-GLP-1. **(C)** The western blot images of the protein levels of GLUT4, p-Akt, and Akt were determined in liver tissue from the mice treated with or without M-GLP-1. The values represent the means ± SD, n = 3–5 per group.
