# Supplementary figures and images for: Engineered Bacteria of MG1363-pMG36e-GLP-1 Attenuated Obesity-Induced by High Fat Diet in Mice
Source: Front Cell Infect Microbiol. 2021 Feb 25;11:595575. doi: 10.3389/fcimb.2021.595575 (PMC7959774; doi:10.3389/fcimb.2021.595575)

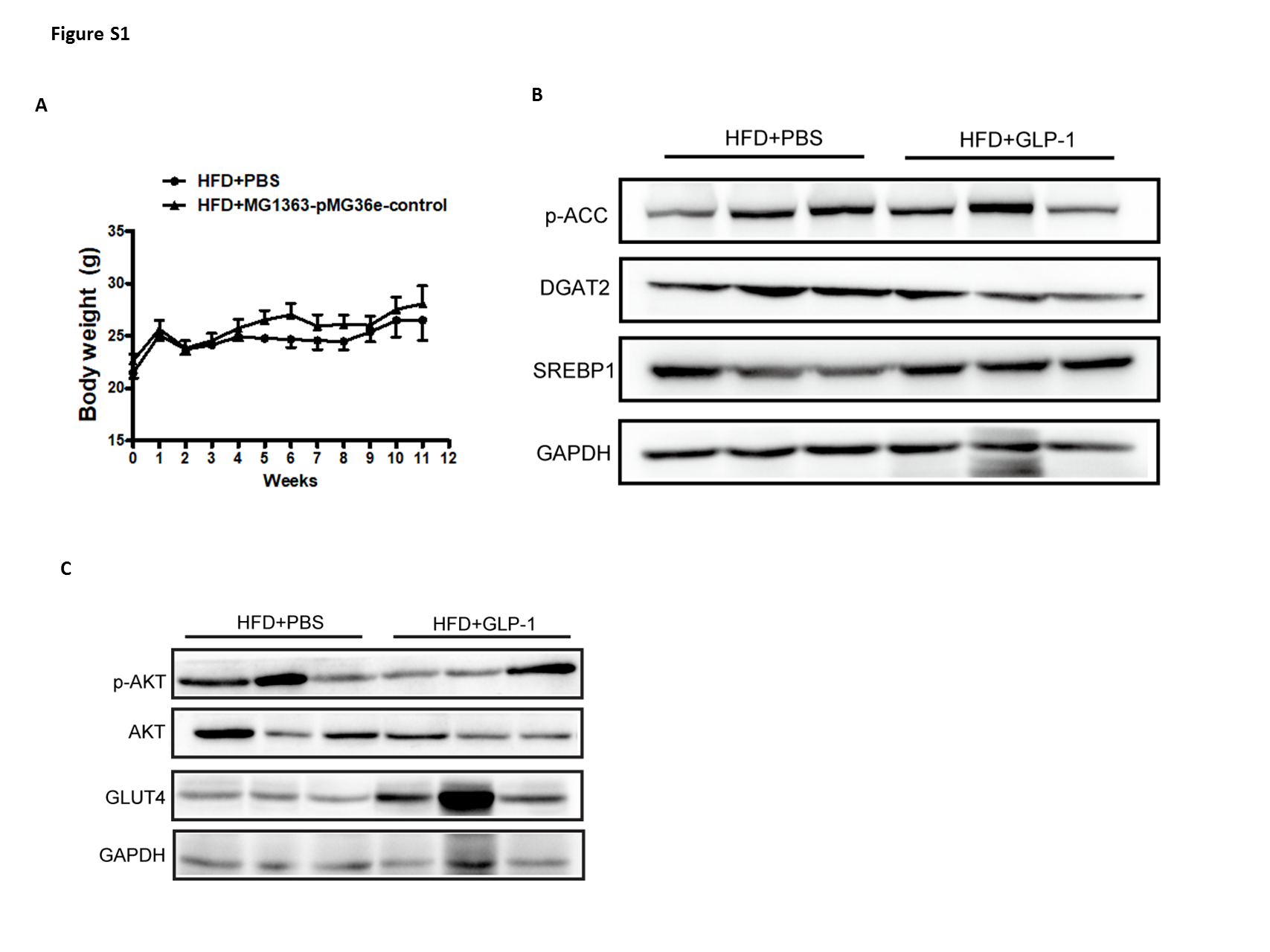

Supplement: Supplementary Figure 1 — (A) The curves of bodyweights in the mice treated with PBS or engineered bacteria but lacking GLP-1 on HFD-fed mice in pre-experiment. (B) The western blot images of the protein levels of SREBP1, DGAT2, and p-ACC were determined in liver tissue from the mice treated with or without M-GLP-1. (C) The western blot images of the protein levels of GLUT4, p-Akt, and Akt were determined in liver tissue from the mice treated with or without M-GLP-1. The values represent the means ± SD, n = 3–5 per group. [file Image_1.tif]
